# Comparison of Biochemical Activities between High and Low Lipid-Producing Strains of *Mucor circinelloides*: An Explanation for the High Oleaginicity of Strain WJ11

**DOI:** 10.1371/journal.pone.0128396

**Published:** 2015-06-05

**Authors:** Xin Tang, Haiqin Chen, Yong Q. Chen, Wei Chen, Victoriano Garre, Yuanda Song, Colin Ratledge

**Affiliations:** 1 State Key Laboratory of Food Science and Technology, School of Food Science and Technology, Jiangnan University, Wuxi, P.R. China; 2 Synergistic Innovation Center for Food Safety and Nutrition, Wuxi, P.R. China; 3 Departamento de Genética y Microbiología (Unidad asociada al IQFR-CSIC), Facultad de Biología, Universidad de Murcia, Murcia, Spain; 4 Department of Biological Sciences, University of Hull, Hull, United Kingdom; Yonsei University, REPUBLIC OF KOREA

## Abstract

The oleaginous fungus, *Mucor circinelloides*, is one of few fungi that produce high amounts of γ-linolenic acid (GLA); however, it usually only produces <25% lipid. Nevertheless, a new strain (WJ11) isolated in this laboratory can produce lipid up to 36% (w/w) cell dry weight (CDW). We have investigated the potential mechanism of high lipid accumulation in *M*. *circinelloides* WJ11 by comparative biochemical analysis with a low lipid-producing strain, *M*. *circinelloides* CBS 277.49, which accumulates less than 15% (w/w) lipid. *M*. *circinelloides* WJ11 produced more cell mass than that of strain CBS 277.49, although with slower glucose consumption. In the lipid accumulation phase, activities of glucose-6-phosphate dehydrogenase and 6-phosphogluconate dehydrogenase in strain WJ11 were greater than in CBS 277.49 by 46% and 17%, respectively, and therefore may provide more NADPH for fatty acid biosynthesis. The activities of NAD^+^:isocitrate dehydrogenase and NADP^+^:isocitrate dehydrogenase, however, were 43% and 54%, respectively, lower in WJ11 than in CBS 277.49 and may retard the tricarboxylic acid cycle and thereby provide more substrate for ATP:citrate lyase (ACL) to produce acetyl-CoA. Also, the activities of ACL and fatty acid synthase in the high lipid-producing strain, WJ11, were 25% and 56%, respectively, greater than in strain CBS 277.49. These enzymes may therefore cooperatively regulate the fatty acid biosynthesis in these two strains.

## Introduction

The interest of microbial oils has increased as they are now used as commercial sources of several nutritionally-important polyunsaturated fatty acids (PUFAs) [1]. Additionally, they are being considered as potential sources of biofuels [2]. Lipid accumulation in oleaginous microorganisms is species- and strain-dependent. Amounts of cellular lipid vary between 20% (w/w) cell dry weight and more than 80% (w/w) [2,3]. Some strains of the oleaginous yeast, *Yarrowia lipolytica* W29, accumulate less than 20% fatty acids of their cell dry weight [4], while *Rhodosporidium toruloides* Y4 accumulates up to 68% lipid of its cell dry weight in fed-batch culture [5]. Among oleaginous filamentous fungi, the lipid content of *Mucor circinelloides* CBS 108.16 and *Mortierella alpina* CBS 696.70 are 25% and 40% of their cell dry weight, respectively, under the same cultivation conditions [6]. Furthermore, lipid in the bacterium, *Rhodococcus opacus* PD630, can account for up to 87% of its cell dry weight [7].

**Fig 1:**
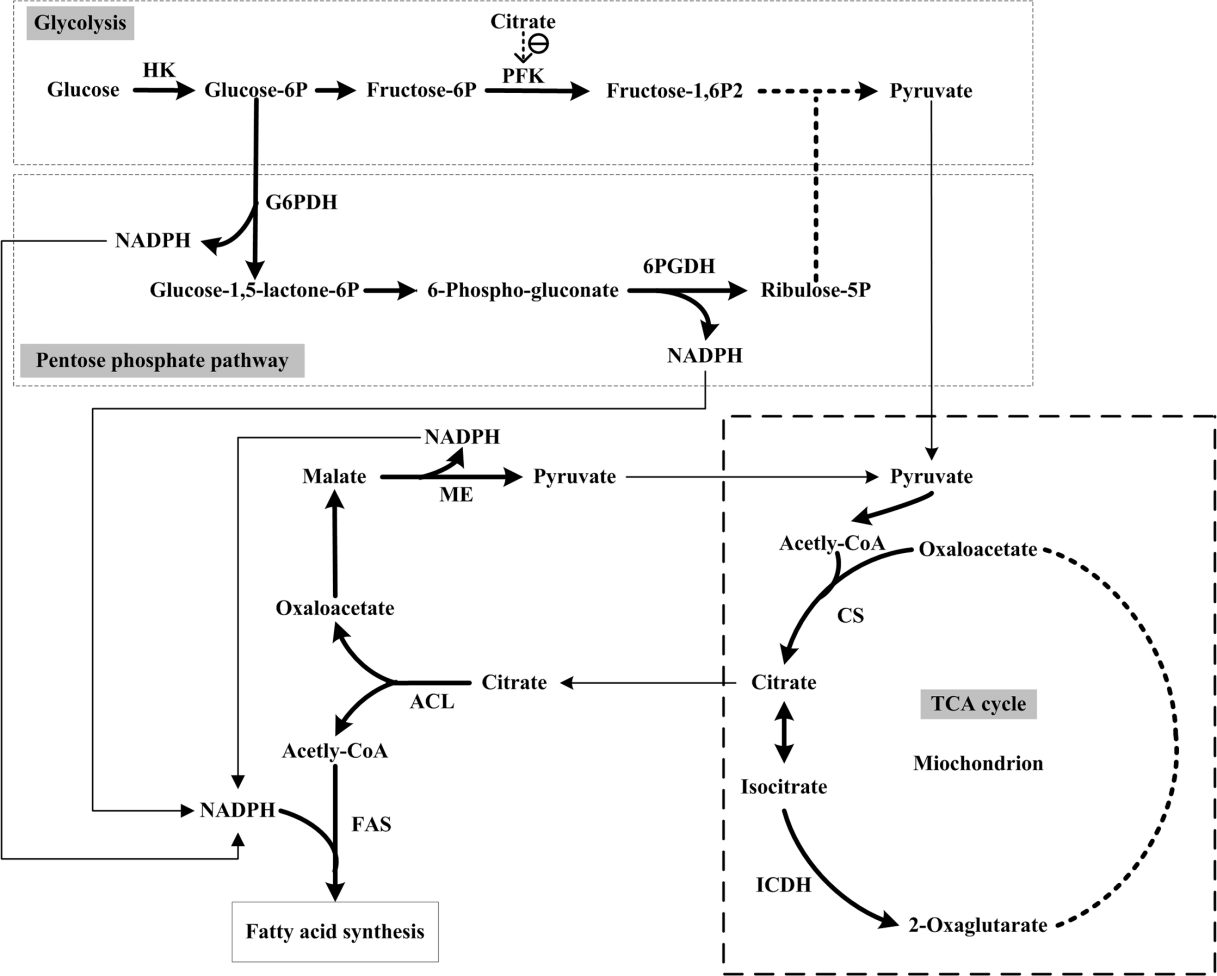
Network of fatty acid biosynthesis in oleaginous eukaryotic microorganisms. HK, hexokinase; PFK, phosphofructokinase; G6PDH, glucose-6-phosphate dehydrogenase; 6PGDH, 6-phosphogluconate dehydrogenase; CS, citrate synthase; ICDH, isocitrate dehydrogenase; ACL, ATP:citrate lyase; ME, malic enzyme; FAS, fatty acid synthase.

The mechanism of fatty acid biosynthesis in oil-rich microorganisms has been extensively studied and we have constructed a network of fatty acid biosynthesis in eukaryotic microorganisms (Fig 1). ATP:Citrate lyase (ACL), which generates acetyl-CoA as the precursor of fatty acids via the cleavage of citric acid, is an essential enzyme for fatty acid biosynthesis in oleaginous yeasts and fungi [3]. Another key process for fatty acid biosynthesis is the provision of reducing power in the form of NADPH needed to reduce acetyl groups (CH_3_-CO-) into the growing acyl chain of a fatty acid (-CH_2_-CH_2_-) [1]. However, the complete molecular mechanism of the different capacity of lipid accumulation among different microbial species, especially in filamentous fungi, is still not clear. It is difficult to decipher the molecular mechanism of lipid accumulation in these different oleaginous microorganisms (e.g., *Y*. *lipolytica*, *M*. *circinelloides*, *M*. *alpina*, *R*. *toruloides*) by comparing the biochemical pathways because of their diverse physiological and biochemical characteristics. Although some comparative genomic studies have been carried out to investigate the molecular mechanism of oleaginicity (lipid-producing capacity) in some microorganisms, no definitive mechanism has yet been established to explain the variation in lipid contents between related species as it is likely that such related species will possess all the relevant enzyme activities for lipid accumulation [8–11].

*M*. *circinelloides*, as an oleaginous fungus, has been used as a model organism to investigate the mechanism of lipid accumulation [6,12,13]. Moreover, it is rich in γ-linolenic acid (GLA, 18:3; cis-6,9,12-octadecatrienoic acid), which is a critical PUFA that may have beneficial effects for prevention and treatment of inflammatory disorders, diabetes, cardiovascular disorders, cancers, atopic dermatitis and some other diseases [3,14]. Indeed, *M*. *circinelloides* was the first microorganism to be used commercially to produce an oil for human consumption—an oil rich in GLA [14]. The strain used, however, only produced 25% of its cell biomass as an extractable oil. We have isolated a strain of *M*. *circinelloides* WJ11 that produces lipid up to 36% (w/w) but the molecular mechanism of the high oleaginicity in this strain is unknown. Moreover, lipid accumulation in another strain of *M*. *circinelloides*, CBS 277.49, whose genome was sequenced by Joint Genome Institute (JGI), was no more than 15% (w/w). Therefore in this study we have compared the key biochemical pathways of *M*. *circinelloides* WJ11 and CBS 277.49 to investigate the differences in molecular mechanisms of lipid accumulation between these strains. To our knowledge, this is the first report of the comparative biochemical analysis of lipid accumulation between two strains within the same species of filamentous fungi.

## Materials and Methods

### Microorganism and cultivation

*M*. *circinelloides* WJ11 was isolated in our laboratory from soil at Jiangnan University. *M*. *circinelloides* CBS 277.49 was also used in this study. 100 μl spore suspension (approx. 10^7^ spores/ml) was inoculated into 150 ml K & R medium [15] held in 1 L flasks equipped with baffles to improve aeration. Cultures were incubated for 24 h at 30°C with shaking at 150 rpm and then used at 10% (v/v) to inoculate 2 L fermenters containing 1.5 L modified K & R medium (2 g diammonium tartrate, 80 g glucose per liter plus inorganic salts). Fermenters were controlled at 30°C with stirring at 700 rpm and aeration at 0.5 v/v min^-1^. The pH was maintained at 6.0 by auto-addition of 4 M KOH or 2 M H_2_SO_4_.

### Determination of cell dry weight (CDW)

Samples were collected periodically from the fermenters for analysis. Biomass was harvested on a dried and weighed filter paper by filtration through a Buchner funnel under reduced pressure and washed three times with distilled water, frozen overnight at -80°C and then freeze-dried. The weight of the biomass was determined gravimetrically.

### Analysis of culture supernatant

Glucose concentration in the culture medium was measured using a glucose oxidase kit. Ammonium concentration in the culture filtrate was determined using the indophenol test [16].

### Analysis of cell lipid and fatty acid composition

Biomass was collected by filtration under reduced pressure, rapidly frozen, and then freeze-dried. Before the extraction of lipid, the freeze-dried biomass was pulverized. Pentadecanoic acid (15:0) was added into the freeze-dried cells as an internal standard and cell lipid was extracted with chloroform/methanol (2:1, v/v) [17]. The extracted cell lipid was methylated with 10% (v/v) methanolic HCl at 60°C for 3 h. The resultant fatty acid methyl esters were extracted with n-hexane and were analyzed by GC equipped a 30 m×0.32 mm DB-WAXETR column with 0.25 μm film thickness. The program was as follows: 120°C for 3 min, ramp to 200°C at 5°C per min, ramp to 220°C at 4°C per min, hold 2 min.

### Preparation of cell extracts

Cell extracts were prepared following the method of Wynn et al [6]. The harvested cells were disrupted by grinding in a mortar with liquid N_2_ and suspended in extraction buffer [100 mM KH_2_PO_4_/KOH, pH 7.5, containing 20% (w/v) glycerol, 1 mM benzamidine∙HCl and 1 mM DTT]. The disrupted cell suspensions were centrifuged at 10000 *g* for 10 min at 4°C and then the supernatants were used for enzyme analysis. Protein concentrations were determined using the method of Bradford with BSA as a standard.

### Determination of enzyme activities

Determination of individual enzyme activity of the cell crude extract was performed according to the published methods with only minor alternations. All enzyme activities were determined using continuous spectrophotometric assays at 30°C. The activities of ATP:citrate lyase (ACL; EC 4.1.3.8), malic enzyme (ME; EC1.1.1.40), glucose-6-phosphate dehydrogenase (G6PDH; EC 1.1.1.49), 6-phosphogluconate dehydrogenase (6PGDH; EC 1.1.1.44), NADP^+^:isocitrate dehydrogenase (NADP^+^:ICDH; EC 1.1.1.42) and fatty acid synthase (FAS; EC 2.3.1.86) were assayed as described by Wynn et al. [6]. The activities of AMP deaminase (AMPD; EC 3.5.4.6), NAD^+^:isocitrate dehydrogenase (NAD^+^:ICDH; EC 1.1.1.41), citrate synthase (CS, EC 2.3.3.1) and phosphofructokinase (PFK; EC 2.7.1.11) were assayed as described by Wynn et al. [12], the activity of hexokinase (HK; EC 2.7.1.1) was assayed as described by Dileepan et al. [18]. Each enzyme activity was measured at three biological replicates to assess reproducibility.

### Statistical analysis

A statistical analysis of the obtained data was carried out using SPSS 16.0 for Windows (SPSS Inc., Chicago, IL). The mean values and the standard error of the mean were calculated from the data obtained from three biological replicates. The differences between the means of the test were evaluated by Student's t-test and *P* < 0.05 was considered as significantly different.

## Results and Discussion

### Cell growth and lipid accumulation with *M*. *circinelloides* WJ11 and CBS 277.49

**Fig 2:**
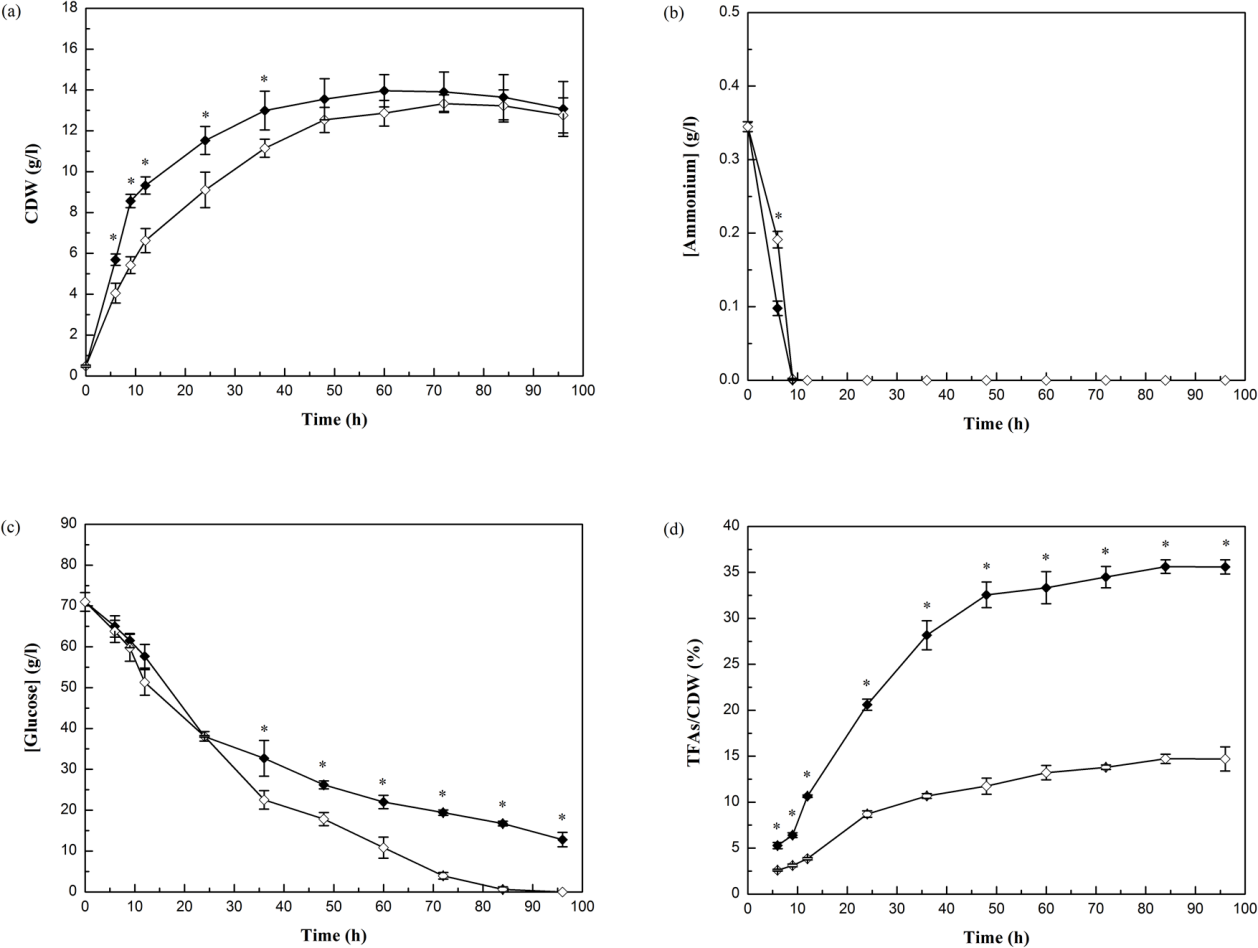
Cell growth and lipid accumulation of *M*. *circinelloides* WJ11 (◆) and CBS 277.49 (◇). (a) Cell dry weight (CDW), (b) ammonium and (c) glucose concentration in culture medium, and (d) total fatty acids (TFAs) content (w/w, TFAs/CDW) of strain WJ11 and CBS 277.49 grown in the modified K & R medium in 2L fermenter were measured. Values were mean of three biological replicates. Error bars represent the standard error of the mean. * *P* < 0.05 significantly different between the two strains.

The cell dry weight (CDW), concentrations of ammonium and glucose in the culture medium, and lipid accumulation of *M*. *circinelloides* WJ11 and CBS 277.49 during growth are shown in Fig 2. Both of strains demonstrated a similar and typical growth profile, and ammonium was used up at approx. 9 h (Fig 2A and 2B). CDW initially increased rapidly during the balanced phase of growth from 0 to 9 h, and then slowed down in both strains after nitrogen exhaustion. The CDW of *M*. *circinelloides* WJ11 was higher than that of strain CBS 277.49 before 48 h. But the CDW of strain WJ11 and CBS 277.49 had no significant difference after 48 h. The maximal CDW was up to 14 g/l in strain WJ11 and that of strain CBS 277.49 was 13.3 g/l. The maximal values of CDW of the two strains are similar to that of another strain *M*. *circinelloides* CBS 108.16 [6].

Glucose was utilized more slowly in *M*. *circinelloides* WJ11 than in CBS 277.49; the remaining glucose concentration in the culture of WJ11 was 12.8 g/l, while it was exhausted in CBS 277.49 after 96 h (Fig 2C). Turnover of storage lipid to yield new biomass typically commences in oleaginous microorganisms when the carbon source is depleted in the cultivation medium [19,20]. However, in our study, the glucose was sufficient during the entire fermentation in both strains. Thus, storage lipid turnover to new biomass would not have occurred in this study.

As Fig 2D shows, from 6 to 9 h, the lipid accumulation in both strains was not significant. After nitrogen depletion from the growth medium, both fungi started to accumulate lipid immediately; from 9 to 48 h, the total fatty acids (TFAs) content increased rapidly from 6.4% to 32.6% in strain WJ11 and from 3.1% to 11.7% in strain CBS 277.49. After 48 h, lipid accumulation slowed. The maximal TFAs in *M*. *circinelloides* WJ11 was 35.6% CDW after 84 h of growth. This was 2.3-fold more than in CBS 277.49 (14.7%). This is in accordance with the previous report which showed *M*. *circinelloides* CBS 277.49 was a strain capable of accumulating no more than 15% (w/w) lipid even under the most propitious conditions [21]. Lipid accumulation in another strain of *M*. *circinelloides*, CBS 108.16, was 20–25% (w/w) [6,22], which is between the amounts in CBS 277.49 and WJ11.

The fatty acid profile of these two fungi are shown in Table 1. The GLA content in TFAs of the low lipid strain, CBS 277.49, (27.1% at 96 h) was almost twice the level compared to the high lipid strain WJ11 (14.5% at 96 h). However, the amount of GLA per gram CDW of strain WJ11 (5.2% at 96 h) was significantly higher than that of strain CBS 277.49 (4% at 96 h) due to its higher lipid production.

**Table 1:** Fatty acid composition (%, w/w of total fatty acids) of *M*. *circinelloides* WJ11 and CBS 277.49 during the fermentation.

Time (h)	14: 0		16: 0		16: 1		18: 0		18: 1		18: 2		18: 3(GLA)		Others	
	WJ11	CBS 277.49	WJ11	CBS 277.49	WJ11	CBS 277.49	WJ11	CBS 277.49	WJ11	CBS 277.49	WJ11	CBS 277.49	WJ11	CBS 277.49	WJ11	CBS 277.49
6	ND	ND	20.7±0.3	21.5±1.4	ND	ND	3.8±0.2	ND	25.6±0.2	14.7±0.6	19.6±0.1	25.5±0.6	30.3±1.1	38.3±1.4	ND	ND
9	ND	ND	18.7±0.1	22.6±0.9	ND	ND	4.9±0.3	ND	31.7±0.4	14.4±0.5	18.3±0	27.0±0.7	26.4±0	36.0±0.8	ND	ND
12	1.1±0	1.2±0.4	19.9±0.2	20.9±2.0	1.2±0.3	ND	6.1±0.3	3.7±0.2	33.3±0.3	17.2±0.5	16.5±0.5	24.7±0.3	20.3±0.2	32.3±1.2	1.6±0.3	ND
24	1.1±0	1.8±0	23.4±0.4	19.8±0.3	0.7±0	1.3±0.1	7.2±0.7	3.1±0	34.6±0.2	21.2±0.3	15.2±0.3	20.6±0.1	14.9±0.5	28.8±0	2.9±0	3.5±0.6
36	1.1±0	1.8±0	24.0±0.2	20.1±0.5	0.8±0	1.6±0	7.4±0.7	2.9±0.1	35.6±0.5	23.0±0.1	14.7±0.4	19.1±0.3	13.6±0.5	27.6±0.3	2.8±0.5	4.1±1.1
48	1.1±0	1.8±0.1	23.8±0.1	20.1±0.6	0.8±0	1.7±0	7.3±0.7	2.6±0.1	36.2±0.6	25.5±0.1	14.6±0.3	18.5±0.2	13.3±0.6	26.4±0.3	2.8±0.7	3.3±0.6
60	1.2±0	1.7±0	23.7±0.1	19.3±0.4	0.8±0.1	2.0±0.2	7.0±0.6	2.5±0.1	37.3±0.9	26.3±0.1	14.7±0.2	18.5±0.3	13.0±0.1	26.4±0.1	2.3±0.7	3.3±0.8
72	1.2±0	1.8±0.1	23.1±0.1	19.0±0.3	0.8±0.1	2.0±0.3	6.5±0.6	2.5±0.1	37.0±0.6	27.2±0.2	14.9±0.2	18.6±0.4	13.8±0.3	26.0±0.3	2.7±0.6	2.9±0.6
84	1.2±0.1	1.7±0	22.4±0.1	18.4±0.5	0.8±0.1	2.0±0.2	6.0±0.6	2.5±0.2	37.2±0.3	27.4±0	15.0±0.2	18.9±0.5	14.6±0.6	26.4±0.4	2.7±0.7	2.8±0.6
96	1.1±0.1	1.7±0	22.1±0.3	18.2±0.5	0.8±0.1	2.1±0.3	5.8±0.5	2.2±0.1	37.8±0.6	27.3±0.2	14.9±0.1	19.2±0.2	14.5±0.2	27.1±0.6	2.9±0.8	2.3±0.3

ND: not detected. GLA: γ-linolenic acid. Others: the fatty acids content < 1% (e.g., 10: 0, 12: 0). All values are mean of three biological replicates ± standard error of the mean.

### Activities of key enzymes related to lipid accumulation in *M*. *ciricinelloides* WJ11 and CBS 277.49

**Fig 3:**
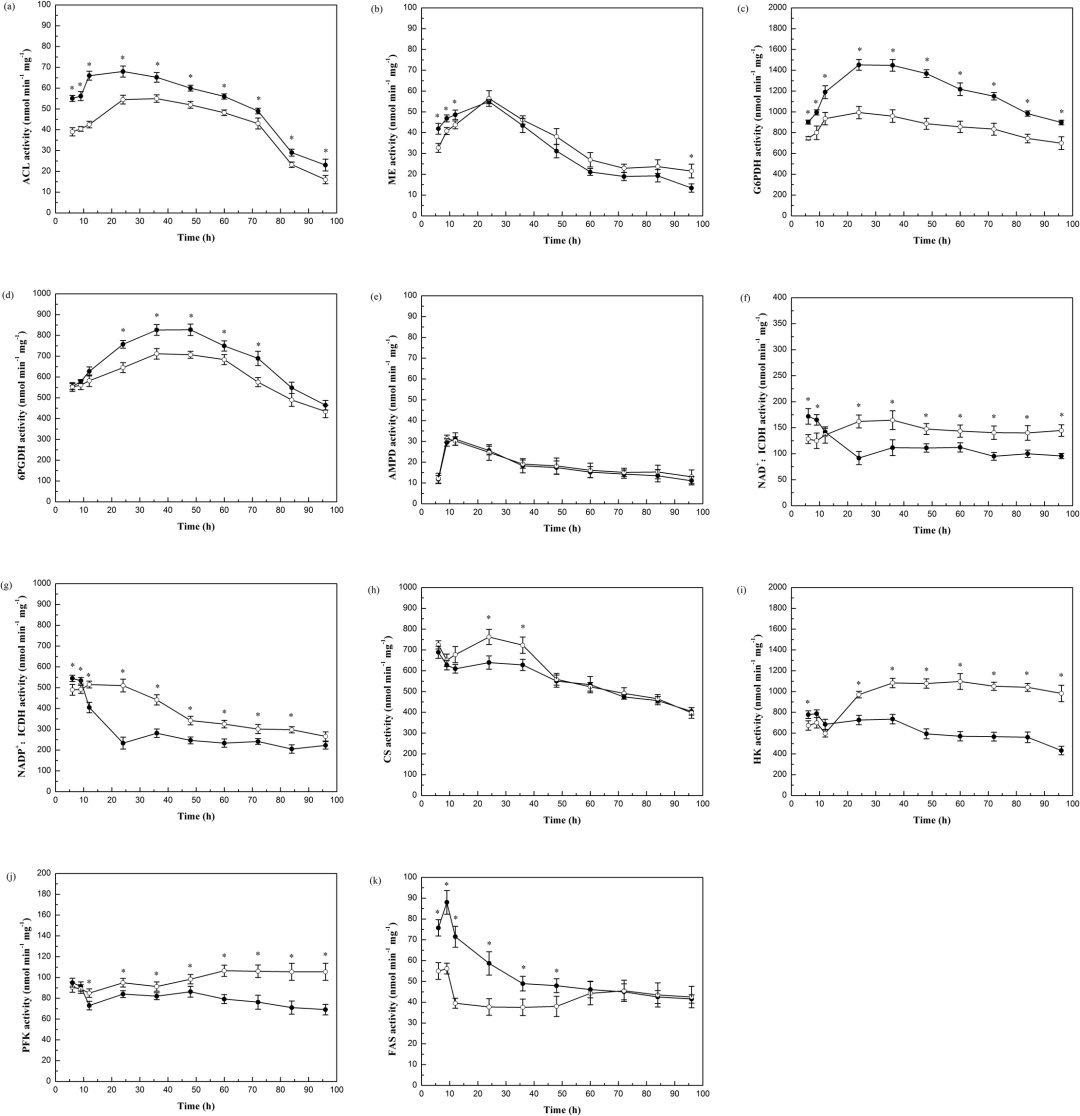
Activity of key enzymes related to lipid accumulation in *M*. *circinelloides* WJ11 (●) and CBS 277.49 (○). Enzymes measured were (a) ACL, (b) ME, (c) G6PDH, (d) 6PGDH, (e) AMPD, (f) NAD^+^:ICDH, (g) NADP^+^:ICDH, (h) CS, (i) HK, (j) PFK, (k) FAS. Values are mean of three biological replicates. Error bars represent the standard error of the mean. * *P* < 0.05 significantly different between the two strains.

To find the potential mechanism underlying the different capacities for lipid accumulation between *M*. *circinelloides* WJ11 and CBS 277.49, the activities of key enzymes involved, or potentially involved, in biochemistry of lipid accumulation were measured for both strains (Fig 3).

Acetyl-CoA is produced in oleaginous microorganisms via ATP:citrate lyase (ACL). In some oleaginous microorganisms, the activity of ACL correlates with the specific rate of lipid synthesis. The enzyme is therefore possibly the rate-limiting reaction for fatty acid biosynthesis in such organisms [23–25]. In this study, the activity of ACL in both strains correlated with the rate of fatty acid biosynthesis (Fig 3A). ACL activity of strain WJ11 was significantly higher than that of strain CBS 277.49, which indicated that more acetyl-CoA, the substrate for fatty acid synthesis, was being provided in strain WJ11 than in strain CBS 277.49, which then might account for the increased lipid production in this strain.

The provision of reducing power in the form of NADPH is another critical process for fatty acid biosynthesis [12]. Previous research has indicated the key role of malic enzyme (ME) to supply NADPH for fatty acid synthesis and desaturation [6,15,26,27]. Furthermore, several studies have suggested that the lipid content of oleaginous microorganisms can be significantly enhanced when ME is overexpressed in some molds or yeasts [28–30]. In this study, the activity of ME in both strains was up-regulated during the initial lipid accumulation phase, and then down-regulated when lipid accumulation slowed down with ME activity being slightly higher in WJ11 than in CBS 277.49 during the initial lipid accumulation phase (Fig 3B). These results are in accordance with recent findings that indicate that the activity of ME was enhanced during lipid accumulation phase compared to the cell growth phase in *R*. *toruloides* and *Trichosporon cutaneum* [10,31].

Besides ME, NADPH can be generated in the cytoplasm by other enzymes, and glucose-6-phosphate dehydrogenase (G6PDH) coupled with 6-phosphogluconate dehydrogenase (6PGDH), as part of the pentose phosphate pathway (PPP), are the prime alternative candidates to supply NADPH for fatty acid biosynthesis [1]. In maize embryos, in the oleaginous microalga, *Chlorella protothecoides* and in the oleaginous yeast, *Yarrowia lipolytica*, the NADPH generated from PPP plays a key role in fatty acid biosynthesis [32–34]. As shown in Fig 3C and 3D, the activities of G6PDH and 6PGDH seemed to be closely associated with lipid accumulation in both strains: they increased when lipid accumulation was increasing, and decreased when lipid accumulation slowed down or stopped. More interestingly, the activities of G6PDH and 6PGDH were significantly higher in high lipid-producing strain WJ11 than that of low lipid-producing strain CBS 277.49, especially in the lipid accumulation phase. This result suggests that the PPP plays a key role in providing NADPH for fatty acid biosynthesis. Thus, the different capacities for lipid accumulation between strain WJ11 and strain CBS 277.49 is possibly due, in part, to the different activities of the dehydrogenases in the PPP.

Oleaginous yeasts, upon nitrogen limitation and therefore at the beginning of lipid accumulation, deaminate AMP to release ammonium and IMP. As NAD^+^:isocitrate dehydrogenase (NAD^+^:ICDH) requires AMP for activity, this loss of AMP causes its activity to rapidly decrease and even to cease completely. Without ICDH activity, isocitrate accumulates and equilibrates back to citrate which is then transported out of the mitochondrion into the cytosol. This citrate then becomes the substrate for ACL and thus provides the increased carbon flux to acetyl-CoA, the substrate for fatty acid synthesis [26]. Nevertheless, the characteristics of NAD^+^:ICDH in oleaginous fungi are distinct from those described for oleaginous yeasts, as these NAD^+^:ICDH are not absolutely dependent on AMP for activity [12,20]. Our results with the two strains of *M*. *circinelloides* show that AMP deaminase (AMPD) activity in both strains increased when the culture became nitrogen-limited and then returned to basal levels. AMPD activity in the two strains had no significant difference (Fig 3E). The activity of NAD^+^:ICDH in strain WJ11, however, rapidly decreased during the lipid accumulation phase. This would then retard the activity of the tricarboxylic acid (TCA) cycle and hence there would be a greater flux of carbon to acetyl-CoA for lipid biosynthesis, thereby resulting in higher lipid accumulation in this strain (Fig 3F).

In contrast, in CBS 277.49, the activity of NAD^+^:ICDH increased, rather than decreasing, during the lipid accumulation phase. In addition, the activity of NAD^+^:ICDH in CBS 277.49 was significantly higher than that of strain WJ11, which suggested a greater flux of carbon through the TCA cycle rather than to acetyl-CoA for lipid biosynthesis, thus, resulting in low lipid accumulation in this strain.

**Fig 4:**
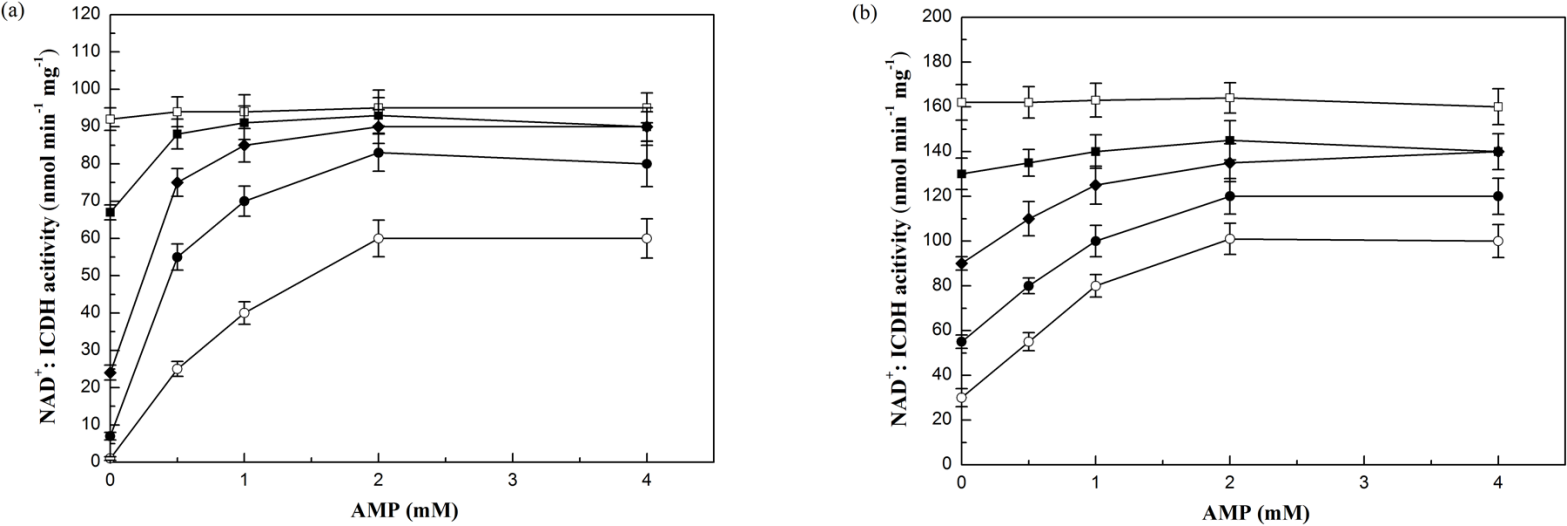
Effect of AMP concentration on NAD^+^:ICDH activity in *M*. *circinelloides* WJ11 (a) and CBS 277.49 (b). The activity of NAD^+^:ICDH of the cell extract at 24 h grown in the modified K & R medium in 2L fermenter was measured, in vitro, in the presence and absence of AMP at different isocitrate concentrations. Isocitrate concentrations: 0.1 mM (○); 0.5 mM (●); 1 mM (◆); 2 mM (■); 5 mM (□). Values are mean of three biological replicates. Error bars represent the standard error of the mean.

The NAD^+^:ICDH activity of these two strains in lipid accumulation phase were also examined at a range of isocitrate and AMP concentrations (Fig 4). The results suggest that NAD^+^:ICDH activity in both strains was not dependent on AMP when the concentration of isocitrate was saturated (5mM). But at low isocitrate concentrations (0.1 to 2 mM), NAD^+^:ICDH activity partially depended on AMP. Furthermore, the NAD^+^:ICDH activity of WJ11 increased more than that of CBS 277.49 when AMP was added, which suggested that NAD^+^:ICDH activity in WJ11 had a higher affinity for AMP compared with strain CBS 277.49. This may partially explain the different activities of NAD^+^:ICDH between the two strains.

The regulation of NADP^+^:isocitrate dehydrogenase (NADP^+^:ICDH) in these two strains was similar to NAD^+^:ICDH (Fig 3G). Indeed, the genomes of the strains WJ11 and CBS 277.49 are predicted to encode a mitochondrial NADP^+^:ICDH. Thus, the activity of NADP^+^:ICDH might also regulate the biosynthesis of fatty acids by affecting the TCA cycle. The rate of TCA cycle was also determined by measuring the activity of the initial reaction, citrate synthase (CS) [35]. The activity of CS in strain CBS 277.49 was significantly higher than that of strain WJ11 in the lipid accumulation phase (Fig 3H). This would suggest that there may be a greater flux of carbon through the TCA cycle in CBS 277.49 and that then diminishes the supply of carbon available for lipid biosynthesis.

The role of hexokinase (HK) in the regulation of glucose uptake had been examined in *Aspergillus niger* and other microorganisms [3]. In our study, the activity of HK of CBS 277.49 was higher than that of WJ11 in the late fermentation period, which may explain why glucose is consumed faster in strain CBS 277.49 than that of strain WJ11 (Fig 3I). Among the various glycolytic enzymes, phosphofructokinase (PFK) is usually involved in the regulation of the glycolytic pathway. As shown in Fig 3J, the activity of PFK in both strains decreased as the ammonium was being consumed. This was in accordance with PFK activity being higher when ammonium is available to the cells [12]. In WJ11, PFK activity decreased when ammonium was depleted and it maintained a lower value during the lipid accumulation phase. This lower PFK activity of strain WJ11 might be partly due to the accumulation of citrate during the lipid accumulation phase as PFK has frequently been reported to be inhibited by citrate [12]. Interestingly, the activity of PFK in CBS 277.49 was higher than that of WJ11 during the lipid accumulation phase, which coincides with it having a more active TCA cycle than WJ11.

These results suggest that CBS 277.49 may have higher activity in glycolytic and TCA cycle activities than WJ11. Thus, in CBS 277.49, carbon flux is being directed to glycolysis and the TCA cycle rather than fatty acids biosynthesis.

De novo fatty acid synthesis is catalyzed by FAS but there are not many reports describing the significance of FAS in regulating the extent of lipid accumulation in oleaginous microorganisms [36]. Nevertheless, in this study, the activity of FAS in strain WJ11 was much higher than that of strain CBS 277.49 in the main growth phase and lipid accumulation phase (Fig 3K) and could explain why more fatty acids are being synthesized in strain WJ11.

### Network of regulation in fatty acid biosynthesis in *M*. *circinelloides* WJ11 and CBS 277.49

In order to understand the possible regulation mechanism of lipid biosynthesis in *M*. *circinelloides* WJ11 and CBS 277.49, the network of regulation in fatty acid biosynthesis during the lipid accumulation phase between the two strains was elucidated according to Fig 1. In WJ11, the decreased activities of HK (by 25%, at 24 h) and PFK (by 12%, at 24 h) may down-regulate glycolysis. Simultaneously, the increased activities of G6PDH (by 46%, at 24 h) and 6PGDH (by 17%, at 24 h) may up-regulate the PPP during the lipid accumulation phase compared with CBS 277.49. This suggests that the glucose flux to PPP is up-regulated in strain WJ11 that increased amounts of NADPH, from G6PDH and 6PGDH activities, for fatty acid biosynthesis. ME has been widely accepted as the critical enzyme to provide the majority of NADPH for fatty acid biosynthesis [1]. In this study, the activity of ME was only slightly higher in WJ11 than in CBS277.49 during the initial part of the lipid accumulation phase, indicating it may play a supplementary role to PPP in providing NADPH for lipid accumulation in WJ11. Compared to CBS 277.49, the activities of NAD^+^:ICDH and NADP^+^:ICDH in WJ11, were decreased (by 43% and 54%, respectively, at 24h), which may retard the TCA cycle, leading to a greater flux of carbon to acetyl-CoA synthesis, and thus increasing fatty acid biosynthesis. In addition, the ACL activity in *M*. *circinelloides* WJ11 was 1.25-fold of that in CBS 277.49 (at 24 h), which further suggested that more acetyl-CoA is produced for fatty acid biosynthesis in WJ11. The activity of FAS was also enhanced (by 56%, at 24h) in WJ11 compared to CBS 277.49 thereby showing why there are greater amounts of fatty acid being synthesized in the former strain.

## Conclusions

*M*. *circinelloides* strain WJ11 isolated in this laboratory produced up to 36% lipid (w/w, CDW), which is higher than that of strain CBS 108.16 (25% lipid, w/w, CDW), a model organism for lipid accumulation studies, and much higher than strain CBS 277.49 (15% lipid, w/w, CDW). The molecular mechanism for the differential lipid accumulation in this fungus was investigated through comparative biochemical analysis of the key pathways involved lipid metabolism. Compared with the low lipid-producing strain, CBS 277.49, in WJ11, the glycolytic pathway was down-regulated while the PPP was up-regulated. ME was also up-regulated. These coordinated regulations thus lead to increased NADPH production. Furthermore, the TCA cycle was retarded, while ACL was up-regulated in WJ11. These changes again will lead to increased production of acetyl-CoA. Lastly, the catalytic machine for fatty acid synthesis was also up-regulated in WJ11. Taken together, the metabolic networks in WJ11 are manipulated to produce more substrate, acetyl-CoA, and more reducing power, NADPH, which are, then, used by a highly active machine (FAS) to produce more lipid.
